# Is Alpha-Synuclein Loss-of-Function a Contributor to Parkinsonian Pathology? Evidence from Non-human Primates

**DOI:** 10.3389/fnins.2016.00012

**Published:** 2016-01-29

**Authors:** Timothy J. Collier, D. Eugene Redmond, Kathy Steece-Collier, Jack W. Lipton, Fredric P. Manfredsson

**Affiliations:** ^1^Department of Translational Science & Molecular Medicine, Michigan State UniversityGrand Rapids, MI, USA; ^2^Hauenstein Neuroscience Center, Mercy Health Saint Mary'sGrand Rapids, MI, USA; ^3^Departments of Psychiatry & Neurosurgery, Yale University School of MedicineNew Haven, CT, USA; ^4^Axion Research FoundationHamden, CT, USA

**Keywords:** alpha-synuclein, Parkinson's disease, non-human primates, substantia nigra, dopamine

## Abstract

Accumulation of alpha-synuclein (α-syn) in Lewy bodies and neurites of midbrain dopamine neurons is diagnostic for Parkinson's disease (PD), leading to the proposal that PD is a toxic gain-of-function synucleinopathy. Here we discuss the alternative viewpoint that α-syn displacement from synapses by misfolding and aggregation results in a toxic loss-of-function. In support of this hypothesis we provide evidence from our pilot study demonstrating that knockdown of endogenous α-syn in dopamine neurons of non-human primates reproduces the pattern of nigrostriatal degeneration characteristic of PD.

## Alpha-synuclein in Parkinson's disease

The best-validated participant in the molecular pathology of Parkinson's disease (PD) is alpha-synuclein (α-syn). Mutations and multiplications of the α-syn SNCA gene locus both produce inherited forms of PD (Polymeropoulos et al., 1997; Singleton et al., 2003; Ibanez et al., 2004). In addition, the presence of α-syn in Lewy bodies and neurites of midbrain dopamine (DA) neurons, the histological hallmark of PD, provides evidence for its association with idiopathic PD (Spillantini et al., 1997). While a pervasive viewpoint postulates that excess α-syn and consequent aggregation directly fuels neurotoxicity in a toxic gain-of-function event, it also is acknowledged that α-syn aggregation may endanger neurons by removing the protein from its normal cellular location and diminishing its function in a toxic loss-of-function event (Perez and Hastings, 2004; Cookson, 2006; Kanaan and Manfredsson, 2012). This issue remains a topic of debate.

Despite this ambiguity, approaches that may indiscriminately reduce α-syn in the central nervous system represent an active area of research as an approach for treating PD (e.g., AFFiRiS PD01, Prothena/Roche PRX002). In this Hypothesis and Theory article, we discuss evidence from rodent and non-human primate experiments suggesting that Parkinson's-like degeneration of the nigrostriatal DA system can be reproduced by eliminating endogenous α-syn from DA neurons vulnerable to degeneration in PD. These studies support the tentative conclusion that α-syn elimination therapies that do not distinguish between native and abnormal forms may compromise the viability of DA neurons and should proceed with caution.

## Knockdown of α-syn in the adult rat results in dose-dependent nigrostriatal degeneration

The lack of overt pathology in α-syn germ-line knockout animals (Abeliovich et al., 2000) and the commonly proposed toxic gain-of-function of the α-syn mutant versions (A53T, A30P, E46K; Kruger et al., 1998; Giasson et al., 2002; Klein et al., 2002; Zarranz et al., 2004) led many investigators, us among them, to pursue targeted knockdown of α-syn expression as a potential therapeutic avenue for PD. However, using recombinant adeno-associated virus (rAAV) expressing a short hairpin RNA (shRNA) to knock down α-syn in mature rat substantia nigra (SN) DA neurons we encountered a surprising result: DA neuron degeneration (Gorbatyuk et al., 2010). In our rodent studies, neurodegeneration could be rescued by co-expression of rat α-syn (rendered insensitive to the shRNA) demonstrating that neuronal loss was explicitly due to a toxic loss-of function of α-syn and not due to non-specific shRNA-mediated toxicity. Moreover, by utilizing several distinct shRNA sequences displaying varying efficiencies of endogenous rat α-syn mRNA knockdown we showed that the extent of neuronal loss was dependent on the level of remaining α-syn. The ability to rescue neurons by co-expression of rat α-syn and the fact that toxicity was proportional to the efficiencies of shRNAs demonstrate that neuronal loss was not due to “off-targeting” of other endogenous mRNAs or due to non-specific shRNA toxicity.

## Knockdown of α-syn in the non-human primate results in dose-dependent nigrostriatal degeneration: A case study

**Figure 1:**
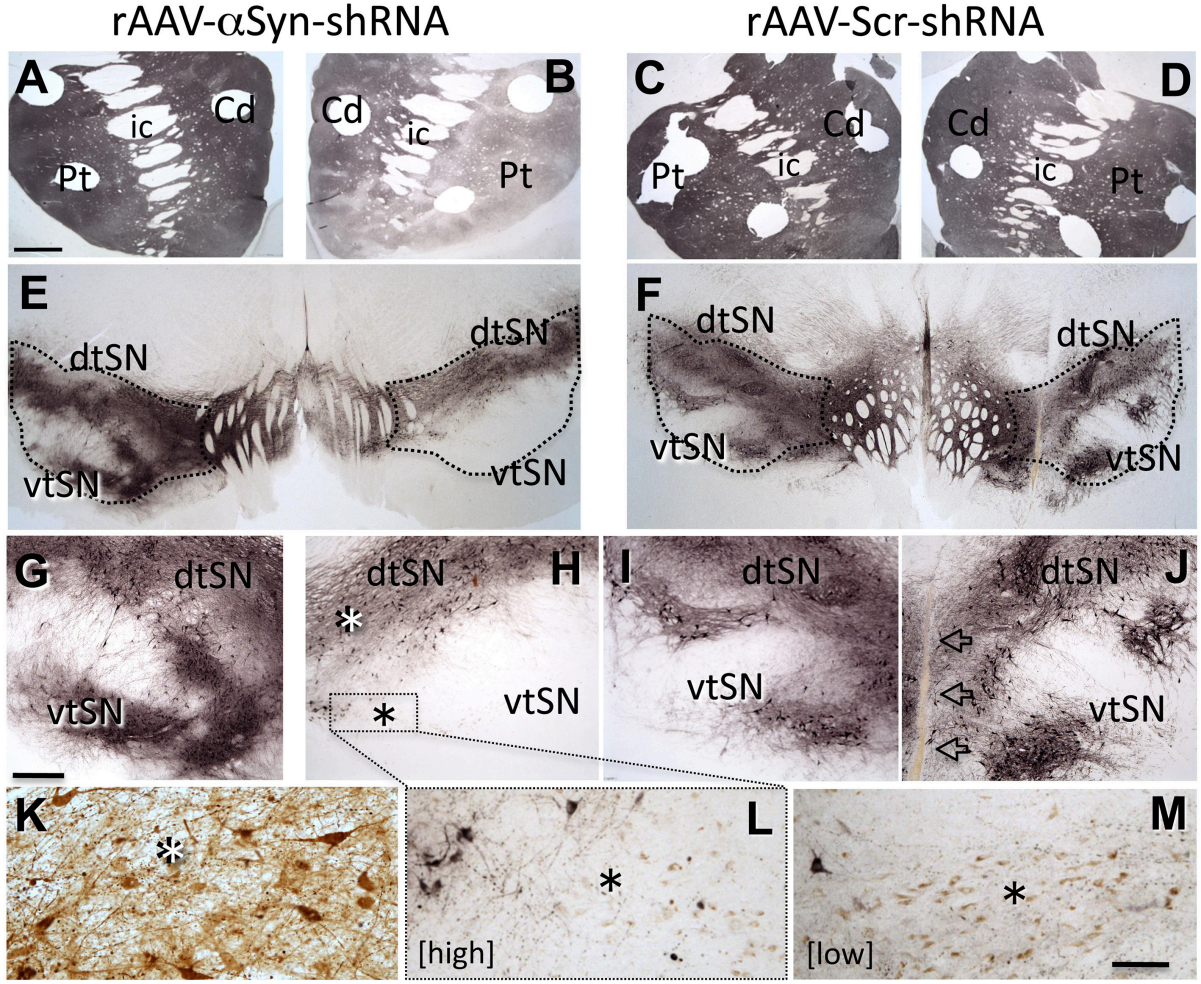
**Knock-down of endogenous α-syn in midbrain dopamine neurons reproduces a PD-like pattern of nigrostriatal degeneration**. rAAV- α-syn-shRNA treatment produces exaggerated degeneration of ventral tier substantia nigra (vtSN) dopamine neurons and denervation of the putamen (Pt) that exceeds that in the caudate (Cd), reproducing the pattern of degeneration observed in Parkinson's disease **[A,B,E,G,H**, transduced hemisphere on right side of micrographs, staining for tyrosine hydroxylase (TH)]. This pattern is not observed with rAAV-scr-shRNA treatment **(C,D,F,I,J**, arrows in **(J)** indicate injection site). Dorsal tier substantia nigra (dtSN) neurons surviving α-syn-shRNA treatment (white asterisk in **H**) were transduced by the vector, but resistant to degeneration (**K**, brown stain for GFP vector tag). While rAAV- α-syn-shRNA of both High and Low titers produced equivalent loss of TH+ vtSN neurons, greater numbers of neuromelanin-positive neurons surviving in this region following Low titer treatment **(M)** indicates phenotype suppression with cell survival, and the low number of these cells in the High titer treatment **(L)** suggests greater overt cell loss [black asterisk in **(H)** indicates vtSN region shown in **(L)** and equivalent region in **(M)** with greater (Low shRNA titer **M**) and lesser (High titer in **L**) numbers of surviving neuromelanin neurons (brown pigment)]. Abbreviations: Cd, caudate nucleus; Pt, putamen; ic, internal capsule; dtSN, dorsal tier substantia nigra; vtSN, ventral tier substantia nigra; Calibration bar in **(A)** = 100 μm and applies to **(A–F)**; bar in **(G)** = 500 μm and applies to **(G–J)**; bar in **(M)** = 100 μm and applies to **(K–L)**.

To begin to further characterize the consequences of knockdown of endogenous α-syn and determine whether effects seen in rats were reproducible in a species more closely related to humans, we generated an α-syn shRNA specific for St.Kitts green monkeys (*Chlorocebus sabaeus*). We injected α-syn shRNA or scrambled shRNA, of two different titers, into the SN of individual monkeys (*N* = 4 total), waited 3 months, and examined DA neuron numbers, morphology, striatal innervation, and striatal DA content. α-Syn shRNA produced region-specific, titer-related, degeneration of SN tyrosine hydroxylase-positive (TH+) neurons and innervation of the striatum, reproducing the pattern of nigrostriatal degeneration observed in PD: SN degeneration was exaggerated in ventral tier neurons (vtSN; Gibb and Lees, 1991) with relative sparing of adjacent ventral tegmental area (VTA) DA neurons, and loss of TH+ fibers in the putamen (Pt) exceeded denervation of the caudate nucleus (Cd) (Kish et al., 1988; Figure 1). Stereologic quantification of TH+ neurons confirmed the qualitative observations: loss of TH+ neurons was greatest in vtSN with the VTA exhibiting relatively little neuron loss. However, the general pattern of TH+ neuron loss was not different in the High and Low titer α-syn shRNA conditions. These neurons also contain neuromelanin, and when counts of neuromelanin-only positive cells were added to the analysis, titer-related differences emerged. The Low titer shRNA condition was associated with the preservation of significantly more neuromelanin-only neurons, suggesting that while loss of TH+ phenotype was equivalent across titers, overt loss of neurons was greater in the High titer condition. The presence of TH-negative, neuromelanin-positive neurons is suggestive of ongoing pathology, an observation that also is seen in early PD. Importantly, co-localization of green fluorescent protein (GFP) as a marker of viral transduction within surviving midbrain DA neurons confirmed that dorsal SN and VTA neurons were transduced, but showed less degeneration (Figure 1). Titer-related differences in striatal DA depletion also were detected. Deficits in DA in the caudate nucleus and putamen were exaggerated in the α-syn shRNA High titer condition and a significant increase in the homovanillic acid (HVA)/DA ratio was only detected in the High titer subject. Increase in the HVA/DA ratio is known to be associated with a compensatory response to ongoing significant degeneration of DA neurons (Zigmond et al., 1990).

## How does loss of presynaptic α-syn contribute to neurodegeneration?

The scope of our one subject per treatment condition pilot study in non-human primates has clear limitations. Nevertheless, the results confirm our previous findings in the rat (Gorbatyuk et al., 2010; Kanaan and Manfredsson, 2012). At this stage our findings, while internally consistent among the treatment conditions, must be considered as generating a hypothesis, rather than being conclusive. While the idea that maintaining α-syn expression is critical for survival of populations of adult neurons has received relatively little investigation, the high prevalence and widespread distribution of α-syn in brain inarguably reflects an important function in synapses. While direct evidence for the loss-of-function hypothesis is in its early stages, many studies provide indirect support for the concept.

Alpha-synuclein exhibits a dramatic redistribution within the neuronal compartment as a function of aging: the best-validated primary risk factor for PD. A quantitative morphological postmortem analysis of α-syn immunoreactivity within SN DA neurons of humans reveals a distinct pattern of increased staining within neuronal soma with advancing chronological age (Chu and Kordower, 2007). Samples were from individuals with a mean age of 31 years (young group, *N* = 6), 55 years (middle-aged, *N* = 4) and 84 years (aged, *N* = 8). Alpha-synuclein immunoreactivity was rarely detected in the soma of SN neurons in young individuals and was restricted to its normal location in the neuropil. With advancing age, a progressive increase in somatic immunoreactivity was observed, culminating in a 639% increase in aged subjects. Quantitation of the intensity of somatic staining on a per neuron basis confirmed this pattern, illustrating a mean 57% increase in aged subjects as compared to young. It is noteworthy that none of these samples exhibited α-syn aggregates, suggesting that this intracellular redistribution of α-syn may be an ongoing aging-related event that precedes processes of aggregation. The same pattern was observed in aging monkeys. Of importance to the loss-of-function hypothesis, no comparison to α-syn distribution/levels in striatum was performed, providing no opportunity to assess whether increased somatic levels are associated with decreased synaptic levels.

Oxidative stress is an established threat to DA neuron viability and can be significantly exacerbated by DA mishandling (i.e., increased cytosolic DA). Indeed, multiple lines of evidence suggest a regulatory role for synuclein in dopamine neurotransmission (Perez et al., 2002; Maries et al., 2003; Wersinger et al., 2003), consistent with the view that loss of presynaptic α-syn may contribute to increased oxidative stress via dysregulation of DA biosynthesis, release and metabolism. Studies in primary cultures of DA neurons demonstrate that in conditions of elevated oxidative stress, endogenous levels of α-syn increase and provide neuroprotection (Quilty et al., 2006; Musgrove et al., 2011). Similarly, transgenic mice over-expressing α-syn are resistant to paraquat-induced toxicity for DA neurons known to be associated with oxidative stress (Manning-Bog et al., 2003). Thus, endogenous α-syn likely provides an important DA neuron defense against oxidative damage.

Mishandling of synaptic DA and the toxic byproducts of its metabolism in the cytosol have a long history of association with the vulnerability of these neurons (e.g., Perez and Hastings, 2004; Segura-Aguilar et al., 2014). Studies of transgenic mice deficient in α-syn, while free of overt DA neuron pathology in adulthood, exhibit changes in DA neurotransmission, with some of these becoming exaggerated in aged animals. Adult, triple knock-out mice for all synuclein family proteins exhibit elevated evoked release of DA in striatum, enhanced turnover and reduced presynaptic DA stores (Anwar et al., 2011). Study of two α-syn deficient mouse lines found evidence for increased stimulated DA overflow in striatum, higher basal extracellular DA levels, decreased expression of the dopamine transporter (DAT) and reduced DA reuptake (Chadchankar et al., 2011). Aged (24–26 months old) α-syn null mice exhibit significant reduction of striatal DA, a decrease in TH+ fibers and decreased striatal levels of TH and DAT (Al-Wandi et al., 2010). These data support the view that α-syn is a pivotal presynaptic regulator of DA neurotransmission and that disruption of this process may result in chronic accumulation of DA in the cytosol.

Perhaps the most direct evidence for an α-syn loss-of-function process related to aggregation comes from the mouse pre-formed fibril (PFF) model of synucleinopathy (Luk et al., 2012). In this model, intracerebral injection of pre-formed fibrils of α-syn seeds misfolding of endogenous α-syn, resulting in progressive accumulation of cytoplasmic aggregates of the protein. In a recent report (Osterberg et al., 2015) the formation of aggregates was tracked over time. The findings indicate that as inclusions mature into a compact form, detection of soluble α-syn shows a parallel decline.

## Is toxicity due to loss of α-syn relevant to Parkinson's disease etiology?

Direct assessment of the relevance of the α-syn loss-of-function hypothesis to PD is a challenge. At present, there is no imaging protocol specific for α-syn, although this is an active area of research. To the best of our knowledge, there has been no study of striatal synaptosome preparations comparing PD to controls and there may be significant technical limitations to this approach with postmortem tissue. In addition, since numerous neuronal circuits project to the striatum, it may be difficult to discern what effect is directly attributable to nigrostriatal projections. Studies of α-syn in body fluids as a biomarker for PD may hold clues, but often are difficult to interpret in the context of entry of α-syn into these compartments and the potential presence or impairment of mechanisms operating to maintain homeostasis. However, α-syn is enriched in brain and blood, and from the context of PD as a “whole body” syndrome it can be argued that a systemic change in α-syn that characterizes PD may be detectable in blood as a reflection of a general process that affects brain. Taken together, biomarker studies targeting α-syn present a set of mixed results (Malek et al., 2014 for review), documenting increases, decreases and no differences in PD subjects as compared to controls. Many of these conclusions likely are linked to differences in sample collection, preparation and the assay employed. One recent report takes a different approach to circumvent the complexities of accurately measuring α-syn protein in blood and measured α-syn transcripts. They analyzed blood samples from three large cohorts of PD patients and controls from prior and ongoing clinical trials individually. All three cohorts supported the finding of an approximate 20% reduction in α-syn transcripts even in newly diagnosed PD patients (Locascio et al., 2015). However, this finding could be interpreted as support of loss-of-function, gain-of-function, or be unrelated to processes operating in brain.

Abundant information from human genetic studies document a clear association between increases in α-syn gene-dosing, or mutations, that exacerbate aggregation and lead to PD. In line with our hypothesis, these all are processes that can directly increase the sequestration of α-syn away from the synapse via increased aggregation kinetics (e.g., enhanced molecular crowding). Nevertheless, one would expect that if α-syn is crucial for maintaining the viability of DA neurons, human genetic data on risk of PD also would implicate mutations or polymorphisms that result in decreased α-syn. Indeed, a recent report has described exactly that (Markopoulou et al., 2014). The dinucleotide repeat REP1 is positioned upstream of the SNCA transcription start site. Polymorphisms at this site are associated with either increased or decreased SNCA expression. Contrary to the investigator's hypothesis that polymorphisms associated with decreased expression of α-syn would be associated with decreased risk of PD, these low α-syn individuals exhibited increased risk of developing PD as well as worse motor and cognitive outcomes.

## Loss-of-function, gain-of-function or a bit of both?

Loss-of-function and gain-of-function hypotheses are not irreconcilable. The absence of an overt parkinsonian phenotype and DA neuron degeneration in germline α-syn knockout mice does not directly address the loss-of-function hypothesis. Whole genome expression analysis of SNCA(−/−) mice identified differential expression of 369 transcripts as compared to wild-type animals (Kuhn et al., 2007). This includes increased expression of transcripts of other synuclein family members, 14-3-3 proteins, TH and neurotrophic factors, decreased expression of pro-apoptotic transcripts, and changes in genes directly involved in vesicle function and neurotransmission. Thus, the lack of a severe phenotype in these animals is likely the result of complex germline compensation for α-syn function. While heritable forms of PD associated with duplication and triplication of the SNCA gene arguably support the toxic gain-of-function hypothesis, germline α-syn overexpression mice do not directly support this contention. The available mouse models exhibit little or no degeneration of SN DA neurons, although similar to α-syn knockout mice, show prominent changes in striatal DA neurotransmission (e.g., Chesselet and Richter, 2011; Crabtree and Zhang, 2012 for reviews). Nevertheless, if the α-syn transgene is induced in the mature animal, nigral degeneration does occur (Lin et al., 2012). Thus, it is plausible that germline compensation, similar to that seen in the knockout, occurs in overexpression transgenic models as well.

While germline transgenic models do little to inform the toxic gain-of-function/loss-of-function debate it is inarguable that too much α-syn and mutant α-syn can produce direct neurotoxicity. *In vitro* studies demonstrate that specific conformations of α-syn exert cytotoxic effects by permeabilizing vesicles, altering calcium flux, impairing mitochondrial function, and inducing apoptosis (Volles et al., 2001; Pieri et al., 2012; Luth et al., 2014; Pacheco et al., 2015). In addition, viral vector-mediated overexpression of α-syn in DA neurons uniformly produces significant, albeit variable in magnitude, neurodegeneration (e.g., Van der Perren et al., 2014 for review). However, in this case it is unclear what precipitates neurodegeneration. Nonetheless, in line with our hypothesis, and with data gained from the PFF model (i.e., Osterberg et al., 2015) it is plausible that loss of soluble endogenous α-syn to aggregation is one contributor to neurotoxicity. Thus, experimental support for both viewpoints suggests a “Goldilocks” biology commonly exhibited by many molecules: too much is bad, too little is bad, enough is just right (Kanaan and Manfredsson, 2012).

## Conclusions

**Figure 2:**
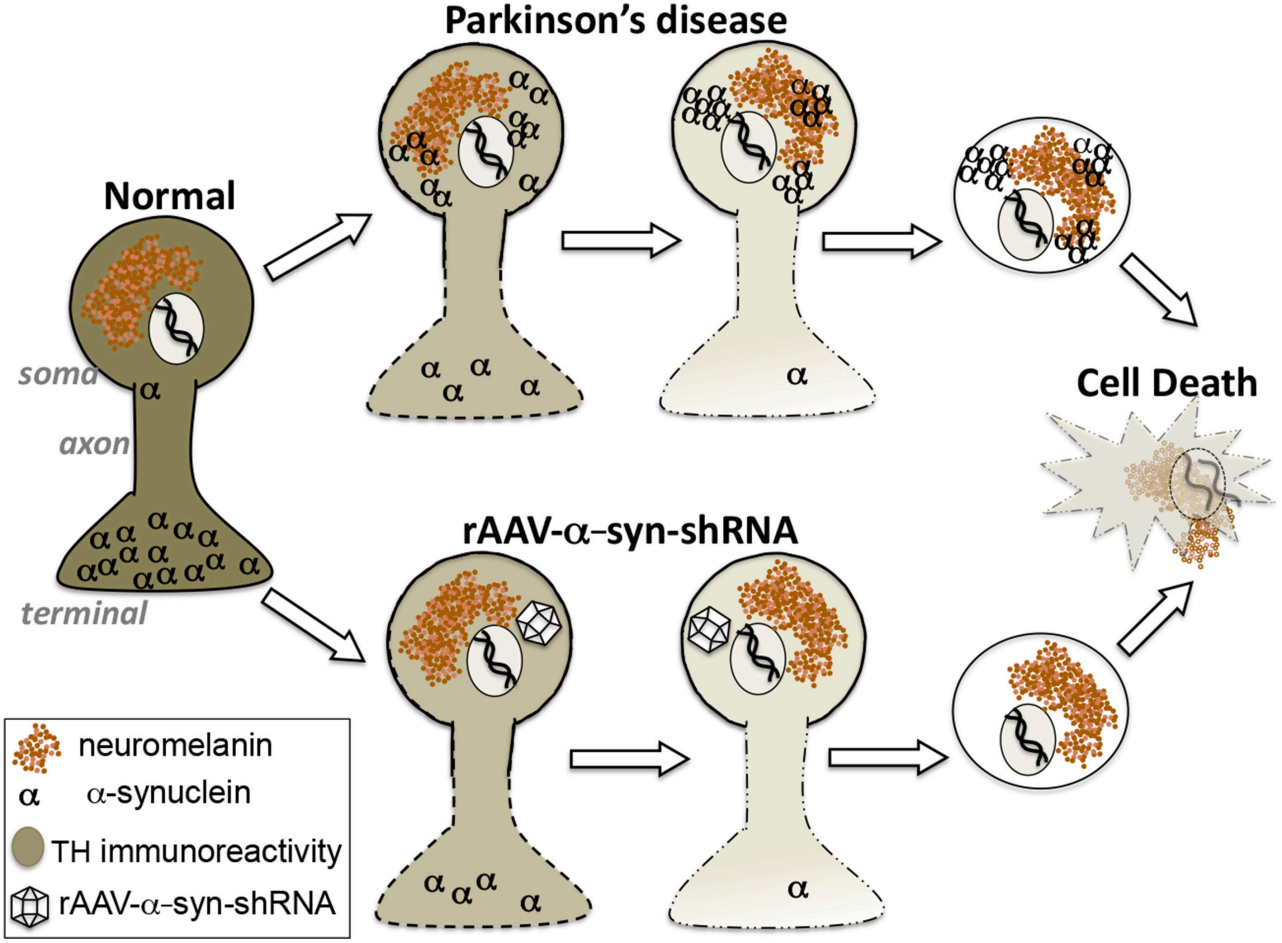
**Hypothesis: Alpha-synuclein loss-of-function as a contributor to parkinsonian pathology**. Our findings suggest that the region-specific degeneration of ventral midbrain dopamine neurons characteristic of Parkinson's disease that is widely attributed to accumulation of toxic aggregates of alpha-synuclein can be accurately reproduced by knockdown of endogenous alpha-synuclein. These differing paths to degeneration converge upon displacement of alpha-synuclein from its natural location at synaptic terminals.

In this Hypothesis and Theory article we address the alpha-synuclein gain-of-function vs. loss-of-function debate as it applies to DA neuron pathology in PD. Our experimental findings in rats and non-human primates, combined with support for the concept from other reports in the literature, leads to the proposal that loss-of-function plays a significant role in this pathology. On its surface, the α-syn loss-of-function hypothesis seems counterintuitive since α-syn accumulation as aggregates, in the form of Lewy bodies, is a key neuropathologic feature of PD. However, while experimental observations largely have been interpreted as suggestive of a cytotoxic role for α-syn, it does not preclude the alternate interpretation that α-syn aggregation reflects processes that prevent α-syn from performing its normal biological functions. Our recent findings in non-human primates support the contention that α-syn loss-of-function in midbrain DA neurons accurately reproduces the pattern of nigrostriatal degeneration observed in PD, and that α-syn aggregation, acting as a “sink,” is one possible driver of this process rather than a direct toxic culprit in cell loss (Figure 2). The findings suggest reconsideration of the relationship of α-syn to PD pathogenesis and caution in the implementation of α-syn clearance therapeutic strategies that do not distinguish between natural forms and pathological forms.

## Author contributions

TC conception, design, acquisition analysis, data interpretation, drafting and revision, DR design, acquisition analysis, revision, KC acquisition analysis, revision, JL acquisition analysis, data interpretation, revision, FM conception, design, acquisition analysis, data interpretation, drafting and revision.

### Conflict of interest statement

The authors declare that the research was conducted in the absence of any commercial or financial relationships that could be construed as a potential conflict of interest.
